# Relationships of Severe Pain and Cognitive Impairment With Acute Care Use in Home Health Patients

**DOI:** 10.1093/geroni/igaa057.800

**Published:** 2020-12-16

**Authors:** Jinjiao Wang, Todd Monroe, Adam Simning, Xueya Cai, Helena Temkin-Greener, Fang Yu, Thomas Caprio, Yue Li

**Affiliations:** 1 University of Rochester, Rochester, New York, United States; 2 The Ohio State University, Columbus, Ohio, United States; 3 University of Rochester Medical Center, Rochester, New York, United States; 4 Arizona State University, Minneapolis, Minnesota, United States

## Abstract

Pain assessment is challenging in patients with cognitive impairment that can lead to inappropriate pain management and unfavorable health outcomes. Using a 10% random sample of Medicare home health (HH) patients ≥ 65 years old from the 2017 Outcome and Assessment Information Set (OASIS) national dataset (N=646,109), we tested the relationships of cognitive impairment and constant, severe pain that interfered with daily living activities with acute care utilization (i.e., hospitalization and emergency department [ED] admission without hospitalization). Patients who had constant, severe, interfering pain (32.57%, N=210,444) were younger, more likely to be female, white, Medicare-Medicaid dually eligible, living alone, and having functional limitations and depressive symptoms, but less likely to have moderate-to-severe cognitive impairment (25.0% versus 31.5%, p<0.001) than others. In multivariable logistic regression models adjusting for the above covariates, when compared with patients with neither cognitive impairment nor severe, constant, interfering pain, those with both conditions were 17% more likely to have hospitalization (Odds Ratio [OR]=1.17, p<0.001) and 13% more likely to have an ED admission without hospitalization (OR=1.13, p<0.001). This was the first study that examined co-occurring pain and cognitive impairment in HH recipients using national OASIS data. Findings suggest that: 1) older HH patients with moderate-to-severe cognitive impairment have lower rates of reported pain that suggests under-recognition; and 2) having severe, interfering pain among cognitively impaired patients significantly increased their risk of acute care utilization. Therefore, systematic protocols and guidelines should be in place to facilitate pain assessment for improved outcomes among HH patients with cognitive impairment.

